# Atypical Dynamic-Connectivity Recruitment in Attention-Deficit/Hyperactivity Disorder Children: An Insight Into Task-Based Dynamic Connectivity Through an fNIRS Study

**DOI:** 10.3389/fnhum.2020.00003

**Published:** 2020-01-31

**Authors:** Stephanie Sutoko, Yukifumi Monden, Tatsuya Tokuda, Takahiro Ikeda, Masako Nagashima, Tsukasa Funane, Hirokazu Atsumori, Masashi Kiguchi, Atsushi Maki, Takanori Yamagata, Ippeita Dan

**Affiliations:** ^1^Hitachi, Ltd., Research & Development Group, Center for Exploratory Research, Tokyo, Japan; ^2^Faculty of Science and Engineering, Applied Cognitive Neuroscience Laboratory, Chuo University, Tokyo, Japan; ^3^Department of Pediatrics, Jichi Medical University, Shimotsuke, Japan; ^4^Department of Pediatrics, International University of Health and Welfare Hospital, Nasushiobara, Japan

**Keywords:** dynamic functional connectivity, an inhibitory control task, functional near-infrared spectroscopy, connectivity states, frontoparietal network, attention-deficit/hyperactivity disorder

## Abstract

Connectivity between brain regions has been redefined beyond a stationary state. Even when a person is in a resting state, brain connectivity dynamically shifts. However, shifted brain connectivity under externally evoked stimulus is still little understood. The current study, therefore, focuses on task-based dynamic functional-connectivity (FC) analysis of brain signals measured by functional near-infrared spectroscopy (fNIRS). We hypothesize that a stimulus may influence not only brain connectivity but also the occurrence probabilities of task-related and task-irrelevant connectivity states. fNIRS measurement (of the prefrontal-to-inferior parietal lobes) was conducted on 21 typically developing (TD) and 21 age-matched attention-deficit/hyperactivity disorder (ADHD) children performing an inhibitory control task, namely, the Go/No-Go (GNG) task. It has been reported that ADHD children lack inhibitory control; differences between TD and ADHD children in terms of task-based dynamic FC were also evaluated. Four connectivity states were found to occur during the temporal task course. Two dominant connectivity states (states 1 and 2) are characterized by strong connectivities within the frontoparietal network (occurrence probabilities of 40%–56% and 26%–29%), and presumptively interpreted as task-related states. A connectivity state (state 3) shows strong connectivities in the bilateral medial frontal-to-parietal cortices (occurrence probability of 7–15%). The strong connectivities were found at the overlapped regions related the default mode network (DMN). Another connectivity state (state 4) visualizes strong connectivities in all measured regions (occurrence probability of 10%–16%). A global effect coming from cerebral vascular may highly influence this connectivity state. During the GNG stimulus interval, the ADHD children tended to show decreased occurrence probability of the dominant connectivity state and increased occurrence probability of other connectivity states (states 3 and 4). Bringing a new perspective to explain neuropathophysiology, these findings suggest atypical dynamic network recruitment to accommodate task demands in ADHD children.

## Introduction

Attention-deficit/hyperactivity disorder (ADHD) is one of the most prevalent neurodevelopmental disorders with continuous increases of prevalence rate (Akinbami et al., 2011; Hill et al., 2015). Due to the elevated demand for shifting the paradigm from qualitative behavior assessments to quantitative biomarker-based evaluations, the approach of neuroimaging (Fu and Costafreda, 2013; Hager and Keshavan, 2015) becomes very attractive for ADHD studies. Previous studies on ADHD suggest hypoactivation in task-dependent regions-of-interest (ROIs) while performing inhibition (Inoue et al., 2012; Monden et al., 2012b; Nagashima et al., 2014b), attention (Nagashima et al., 2014a,c), working memory (Ehlis et al., 2008), and verbal fluency (Schecklmann et al., 2008) tasks. Furthermore, ADHD subjects revealed abnormalities on the default mode network (DMN) and the sensory-motor network in a resting state (Castellanos et al., 2008; Choi et al., 2013), impaired the cingulo-frontoparietal (CFP) network and the cortico-striato-thalamocortical circuitry associated with attention (Bush, 2010, 2011) and more complex executive functions (Castellanos et al., 2006). ADHD adolescents showed weak connectivities within the inhibition network and failed to suppress irrelevant networks (e.g., DMN) during response inhibition (van Rooij et al., 2015). Both activation and network characteristics had been proposes as screening biomarkers (Monden et al., 2015; Uddin et al., 2017).

The conventional functional connectivity (FC) concept assumes static connectivity over temporal courses (Biswal et al., 1995). However, the assumption of stationarity dismisses the complexity of brain processes. FC analysis was progressing from concepts of static to dynamic temporal networks which several connectivity states were found to alternate (Liu and Duyn, 2013). Dynamic FC analysis provides the information of shifting connectivity states that are not addressed in the static FC analysis (for reviews, see Hutchison et al., 2013; Preti et al., 2017). The connectivity state depicts connectivity patterns, such as strong or weak associations between brain regions. For example, a connectivity state represented a positive correlation between posterior cingulate cortex (PCC) and anterior cingulate cortex (ACC) together with a negative correlation between PCC and right inferior frontal operculum. Furthermore, another connectivity state revealed the opposite correlation characteristics (Chang and Glover, 2010). Connectivity states and its characteristics are task-dependent that reveal distinct differences between resting-state (RS) and cognitive tasks (Gonzalez-Castillo et al., 2015; Gonzalez-Castillo and Bandettini, 2018). In order to accommodate cognitive flexibility, reconfiguration of frontal networks were evidenced by dynamic FC analysis (Braun et al., 2015). In addition, the connectivity state is one of essential information related to the pathophysiology of disorders; dynamic FC-based biomarkers for schizophrenia (Damaraju et al., 2014) and Alzheimer’s disease (AD; Jones et al., 2012) may potentially surpass the benefits of static FC-based biomarkers.

Even though fMRI offers high spatial resolution (in the order of square millimeters) and is a well-established analysis method (Bandettini, 2012), another neuroimaging technique, namely, functional near-infrared spectroscopy (fNIRS; Maki et al., 1995; Strangman et al., 2002; Boas et al., 2014), provides better temporal resolution and motion tolerance (Hoshi, 2005). A critical issue facing fMRI is difficulty in measuring infants and children under an unnatural environment (i.e., a confined and noisy space that brings the risk of anxiety and claustrophobia; Byars et al., 2002). Measuring children with abnormal symptomatic behaviors is a challenging task for fMRI because of susceptible motion artifacts, which lead to low rates of successful measurement (Vaidya et al., 1998). For future clinical translational purposes, therefore, application of fNIRS in pediatric studies is inevitable. Several studies related to neurodevelopmental disorders [e.g., ADHD (Monden et al., 2012a), autism spectrum disorder (Ikeda et al., 2018a)] have been pursued using fNIRS.

FC analysis using fNIRS has been investigated, and the results of those investigations revealed that fNIRS detects similar networks to networks distinguished by fMRI (Duan et al., 2012; Sasai et al., 2012; Tak et al., 2015) and also achieves high reliability and reproducibility (Niu et al., 2011, 2013; Zhang et al., 2011). Furthermore, fNIRS is a valid method for quantifying characteristics of RS dynamic FCs (Li et al., 2015). RS dynamic FCs detected by fNIRS also revealed abnormalities of connectivity states in mild-cognitive-impairment and AD patients (Niu et al., 2019). The RS task was mainly studied due to its low task demand. However, test-retest reliability of the RS task was found to be insufficient (Lang et al., 2014) due to unavoidable ongoing cognitions at rest, such as inner experiences (i.e., speaking, seeing, thinking, sensory awareness, and feeling; Doucet et al., 2012; Hurlburt et al., 2015). Therefore, the task-based measurement with less-variant and controlled task-positive networks may be a potential approach. Task-based networks drawn from fNIRS measurements have been evaluated for an ADHD screening biomarker and showed a promising result of 88% accuracy (Sutoko et al., 2019a). However, to date, fNIRS has not been applied to study task-based dynamic FCs.

The current study, therefore, aims to explore and evaluate the feasibility of task-based dynamic FCs (measured by fNIRS) in particular data of typically developing children (TD) and children with ADHD during a task, such as an inhibition control task. The application of FC analysis using fNIRS to children also emphasizes the benefits of fNIRS measurement over less-practical fMRI measurement. By using task-based dynamic FC analysis, we addressed three questions: (1) Is shifting brain connectivity observed by fNIRS during the temporal task course? (2) How connectivity states within measured regions (i.e., ROIs) shift (and how frequently) during the temporal task course? While previous studies focus on particular networks over temporal courses, the dynamic FC analysis takes into account all networks within measured regions for categorizing them in connectivity states. This analysis becomes more robust compared to the fluctuation of connectivity strength during the temporal course. (3) What are the differences between TD and ADHD children in dynamically shifted connectivity states? Compared to the static FC analysis, the dynamic FC analysis is capable to resolve the mechanism of shifting connectivity state in response to the task. Brain connectivity states expectedly change during the temporal task course, and the changes may phase-lock with stimulus information (e.g., onset and end of the stimulus). We hypothesized that several connectivity states suggesting task-related and task-irrelevant networks may be alternated with different probabilities of occurrence. Brain-network impairment in ADHD subjects (as mentioned above) may also occur in atypical connectivity states and/or dynamic occurrence probabilities as implications of task demands. These results can provide ADHD neuropathophysiology from a noteworthy perspective and bring insights for clinical purposes.

## Materials and Methods

### Subjects and Experimental Design

Subject data described here originally came from two different previously reported datasets: (1) 21 medication-naïve ADHD-children data (right-handed; 17 boys; 7.8 ± 1.7 years old; Tokuda et al., 2018; Sutoko et al., 2019b); and (2) 21 age-matching subjects selected from 30 TD control data (right-handed; 15 boys; 8.5 ± 1.8 years old; Monden et al., 2015). Both the TD and ADHD children were evaluated according to the Wechsler Intelligence Scale for Children—Third Edition (WISC-III). Full-scale IQ scores of the TD (105.5 ± 12.3 of IQ) and ADHD (92.8 ± 12.9 of IQ) groups were unmatched (two-sample *t*-test). The TD group had higher (*p* < 0.01; *t*_(40)_ = 3.27) full-scale IQ scores than those of the ADHD group. All subjects provided oral consent, and written consent was obtained from all subject’s guardians according to the latest version of the Declaration of Helsinki. Previously related and current studies were approved by the Ethics Committees of Jichi Medical University Hospital, the International University of Health and Welfare, Chuo University, and Hitachi, Ltd. This study is collaboration research between Jichi Medical University Hospital, the International University of Health and Welfare, Chuo University, and Hitachi, Ltd. An ADHD-subject was excluded from further analysis because of an unexpected technical problem in data saving.

All subjects performed a visual inhibition control task. The block-design paradigm is described in detail elsewhere (Monden et al., 2012b; Nagashima et al., 2014b; Ikeda et al., 2018b). The paradigm involved two types of task blocks, namely, Go and Go/No-Go (GNG) blocks. The Go block was performed seven times and the GNG block was performed six times in turns in which the Go block always preceded the GNG block (i.e., baseline). In brief, the subjects were required to respond to any visual stimulus (e.g., tiger or lion pictures; 800 and 200 ms for display interval and between-picture waiting interval, respectively; 24 times) by pressing a designated button during the Go block. However, the subjects were asked to control their response and react only on selective visual stimulus (e.g., giraffe picture; 800 and 200 ms for display interval and between-picture waiting interval, respectively; 24 times with 50% occurrence of the selective stimulus) during the GNG block. The 3-s instruction was displayed prior to the Go and GNG blocks in order to guide the subjects in performing upcoming blocks (e.g., press the button for tiger or lion; do not press the button for elephant). This task lasted for about 5–6 min. Even though a previous experiment on a medication-naïve ADHD dataset adopted a randomized, double-blind, placebo-controlled, and crossover study, the current analysis incorporated only pre-administration data (neither medication nor placebo) in order to focus only on the nature of ADHD characteristics.

As conducting the measurement, three parameters of behavioral performance, such as accuracy of selective stimulus-response, accuracy of non-selective stimulus-response, and response time of selective stimulus-response, were measured. Behavioral performances have been reported in our previous publications. Inconsistent behavioral characteristics were found in term of group comparison (i.e., TD vs. ADHD; Monden et al., 2012b; Nagashima et al., 2014b). Despite these behavioral inconsistencies, hypoactivation in the right middle frontal gyrus (MFG) and inferior frontal gyrus (IFG) was constantly observed. In summary, the interpretation of behavioral performances is confounding, and brain dysfunctions prompt better understandings for inter-group differences. Consequently, the relationships between behavioral performances and brain parameters (i.e., connectivity states) were not subjected to the current analysis.

### fNIRS Measurement

**Figure 1:**
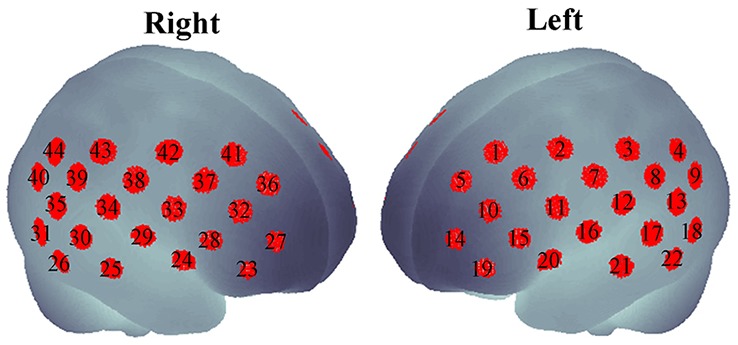
Forty-four channels spatially registered on bilateral hemispheres.

As the subjects were performing the task, a dual-wavelength fNIRS system (695 and 830 nm; ETG-4000, Hitachi Medical Corporation, Tokyo, Japan) was used to measure cerebral hemodynamic changes. Eight emitters and seven detectors were incorporated in a probe (in a 3 × 5 arrangement). The two probes were placed on the head of the subject according to the placement as described elsewhere (Monden et al., 2012a). The probe placement aimed to cover the bi-hemispheric lateral prefrontal-to-inferior parietal lobes (Garavan et al., 1999; Liddle et al., 2001; Rubia et al., 2003; Herrmann et al., 2004, 2005). The measured regions (also called “channels”) were approximately estimated at the midpoint between an emitter and a detector. Therefore, measurements by 44 channels could be simultaneously obtained from the two probes. Emitter and detector positions was measured using a 3D digitizer, and the channel locations were spatially registered in the Montreal Neurological Institute (MNI) standard brain space by the probabilistic registration method (Tsuzuki et al., 2007, 2012; Tsuzuki and Dan, 2014) as shown in Figure 1.

### Signal Processing

**Figure 2:**
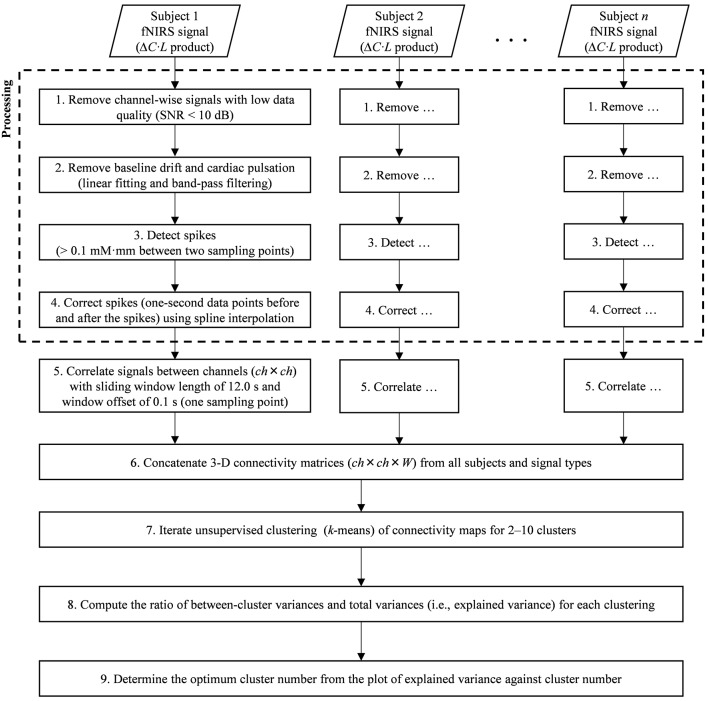
Flow chart of signal processing (dashed square) and dynamic-functional-connectivity (FC) analysis.

**Figure 3:**
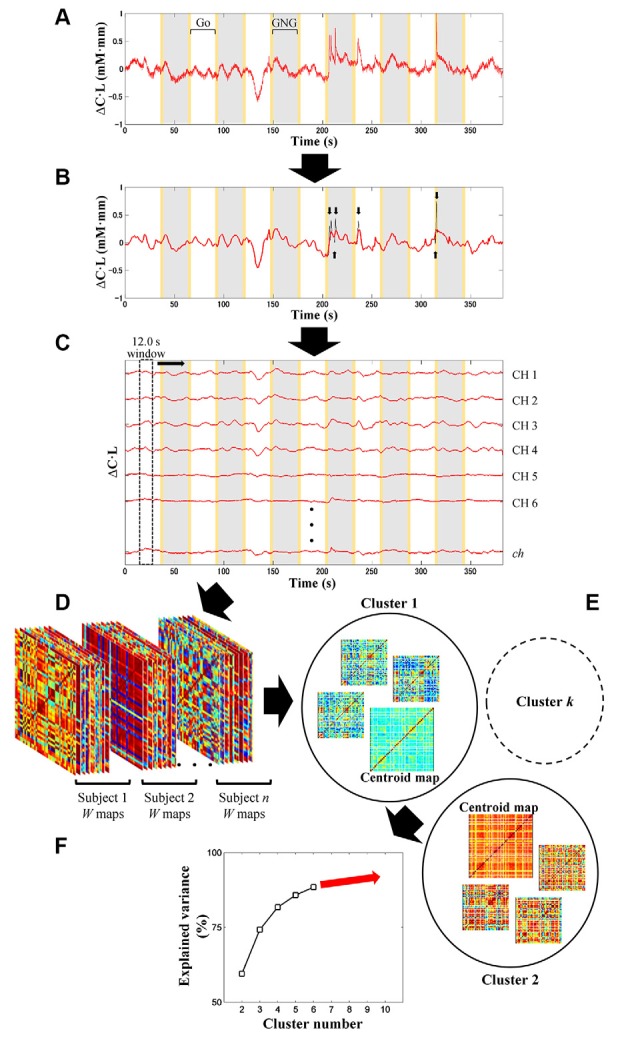
Illustration of signal preprocessing **(A,B)** and dynamic-FC analysis **(C–F)**. Signal processing was applied to $\Delta C_{{\text{O}}_{2}\text{Hb}}\cdot \text{L}$ and *ΔC*_HHb_·L signals (i.e., signal type) of each subject and channel (*ch*) **(A)**. After linear fitting and band-pass filtering (5th order of Butterworth filtering; 0.01–0.8 Hz), motion artifacts with spike characteristics (black plot) were detected (black arrows) and corrected by spline interpolation **(B)**. Gray and yellow patches represent the Go/No-Go (GNG) block as stimulus intervals and the instruction intervals, respectively. Sliding-window-correlation analysis with 12.0-s window length and 0.1-s window offset between channels for each subject and signal type resulted in *W* (total measurement minus window lengths; in points) number of *ch* × *ch* maps **(C)**. Maps (*ch* × *ch* × *W*) obtained from all subjects and signal types were concatenated **(D)**. Cluster (i.e., connectivity state) number was optimized afterward (starting from 2 to 10 clusters) by *k*-means clustering **(E)**. In each clustering, a parameter called explained variance was computed. To determine the optimum cluster number, the relationship between cluster number and explained variance was then plotted **(F)** by using the elbow assessment method.

fNIRS signal processing was performed on the MATLAB-based software Platform for Optical Topography Analysis Tools (POTATo, Hitachi, Ltd, Research and Development; Sutoko et al., 2016). Optical density of transmitted NIR light was detected for two signal types, oxygenated and deoxygenated hemoglobin (O_2_Hb and HHb) and converted into the product of hemoglobin-concentration change and optical-path length ($\Delta C_{{\text{O}}_{2}\text{Hb}}\cdot \text{L}$ and *ΔC*_HHb_·L in mM·mm units) according to the modified Beer-Lambert law (Delpy et al., 1988; Maki et al., 1995). A flow chart of the analysis of fNIRS data is summarized in Figures 2, 3. The signal-processing steps are specified inside the dashed box in Figure 2. Signal processing was applied to $\Delta C_{{\text{O}}_{2}\text{Hb}}\cdot \text{L}$ and *ΔC*_HHb_·L signals of each subject and channel (Figure 3A). Channel signals with signal-to-noise ratio (SNR; power ratio of 0.01–0.15 Hz to 4.5–5 Hz) less than 10 dB were eliminated (about 5% of the total number of signals). To remove baseline drift and cardiac pulsation, basic processing including first-degree polynomial fitting and band-pass filtering (5th order Butterworth filtering at 0.01–0.8 Hz) was applied to remaining signals (92.0 ± 10.3% across subjects). Furthermore, sudden changes (> 0.1 mM·mm between two sampling points), also called “spikes” (i.e., a motion-artifact characteristic), were assessed. Data points affected by the spikes (together with one-second data points before and after the spikes) were then removed from a continuous signal (1.11 ± 1.69% of total data points across subjects), and the removed data points were estimated using cubic spline interpolation (third-order polynomials). An example result of spike removal and correction is shown in Figure 3B.

### Analysis of Dynamic Connectivity

The five steps of the dynamic-connectivity analysis adapted from the sliding-window correlation (SWC) method (Hutchison et al., 2013; Preti et al., 2017), which follows the signal-processing steps shown in Figure 2, are detailed below.

In the first step, the channel-wise processed signals are correlated to each other (*ch × ch*) with a window length of 12.0 s and window offset of one sampling point (0.1 s; see Figure 3C). The greater correlation coefficients (*r*) are computed, the stronger connectivities between regions are interpreted. The total number of connectivity maps (i.e., *W*) for each subject and signal type is given by the total data points (5–6 min; 3,000–3,600 points) minus the sliding-window points (12.0 s; 120 points). To monitor the transition of dynamic connectivity from baseline to task and vice versa, fifty percent of the stimulus interval (GNG block) was selected as the sliding window. Using a longer sliding window than the stimulus interval will neglect subtle connectivity changes during transitions.

In the second step, dynamic-connectivity maps obtained from all temporal points, subjects, and signal types are concatenated in 3D matrices (as shown in Figure 3D).

In the third step, because the number of connectivity states involved in the task is not presumed, it is determined by trial-and-error unsupervised clustering for all connectivity maps (starting from 2 to 10 clusters) by using the *k*-means clustering (Figure 3E). Each cluster produces a centroid map. Centroid maps characterize attribute centers with the shortest total Euclidean distances of within-cluster members to the centroids. Those clusters represent connectivity states. Low-SNR signals (which had been eliminated beforehand) formed incomplete connectivity maps, which were therefore temporarily rejected during the trial-and-error clustering.

In the fourth step, to evaluate the correctness of clustering, a parameter called “explained variance” is calculated. The explained variance (given as a percentage) indicates the ratio of between-cluster variances and total variances. As the cluster number increases, the between-cluster variance decreases, and the explained variance reaches approximately 100% (Figure 3F). Cluster number should be appropriately selected; that is, excessively low and high cluster numbers would result in mismatched clustering and overfitting, respectively.

In the fifth step, the elbow method was used to determine a threshold for cluster number, which, if surpassed, clustering is not effectively improved (Tibshirani et al., 2001). The threshold was then defined as the optimum cluster number (i.e., connectivity states). Thereafter, several centroid maps were obtained by clustering with the optimum cluster number. The incomplete connectivity maps previously disregarded were approximately classified into specific clusters according to the highest similarity among the remaining characteristics of connectivity maps and centroid maps.

### Statistical Analysis

**Figure 4:**
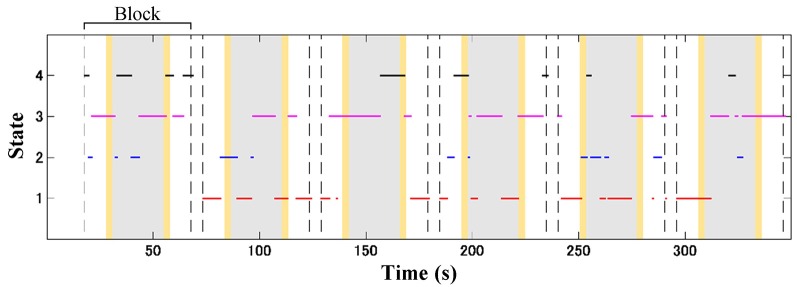
Illustration of dynamic shifting between four connectivity states (red, blue, magenta, and black lines for connectivity states 1, 2, 3, and 4, respectively) in an attention-deficit/hyperactivity disorder (ADHD) child (a 7-years old boy) during the GNG task. Gray patches represent the GNG block as stimulus intervals, and yellow patches denote the instruction intervals. A block interval is specified by 13, 24, and 13 s of the baseline (10 s of Go block and 3 s of instruction), stimulus, and post-stimulus (10 s of Go block and 3 s of instruction) intervals, respectively. In one time measurement, six-block intervals were acquired according to the number of stimuli.

Subject-wise connectivity maps with labeled clusters were compartmentalized according to the block interval (13, 24, and 13 s) of the baseline (10 s Go block and 3 s instruction), stimulus (GNG block), and post-stimulus (3 s instruction and 10 s Go block) intervals. The temporal information of the connectivity map was aligned to the onset of the connectivity window. The inter-stimulus interval was 30 s (including 24 s for the Go block and two 3-s instructions); thus, the connectivity maps in the post-stimulus and baseline intervals were unlikely to overlap. Figure 4 shows an illustration of dynamic change for four connectivity states from an ADHD child over the temporal course. Subject-wise probability occurrence was defined as the probability of specific connectivity states occurring during six blocks. Because the occurrence probability follows the discrete probability distribution, statistical tests were performed according to non-parametric manners. Kruskal–Wallis *H* test was applied to median values of occurrence probability in order to evaluate the effect of connectivity state. The effect of connectivity state and signal type on median values of occurrence probability was determined by using Friedman test. Dunn-Šidák *post hoc* analysis was applied to significant effects given by the Kruskal–Wallis *H* test. Furthermore, the effect of signal type on median values of occurrence probability for each connectivity state was evaluated by using the Wilcoxon signed-rank test. The difference between the TD and ADHD groups (i.e., the effect of the group) in terms of occurrence probabilities of (O_2_Hb and HHb) connectivity states was statistically analyzed by using Wilcoxon rank-sum test.

## Results

### Selection of Optimum Cluster Number

**Figure 5:**
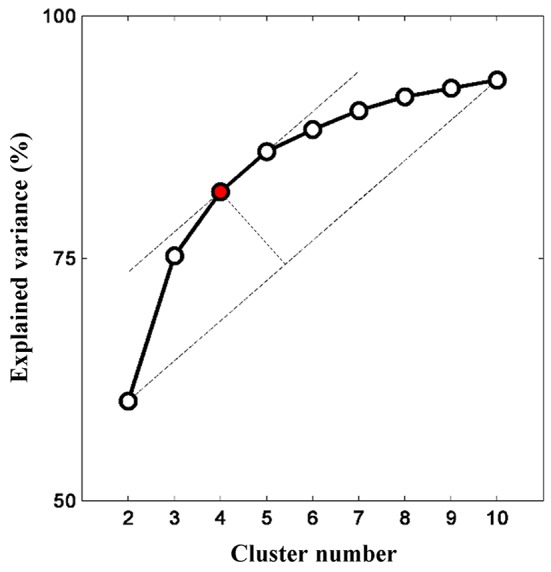
Relationship between cluster number and explained variance. Increasing cluster number consequently increases the explained variance (given as a percentage). The explained variance gradually plateaus (or insignificant increase) as more clusters are added. The optimum cluster number is four clusters as determined by the elbow assessment method.

**Figure 6:**
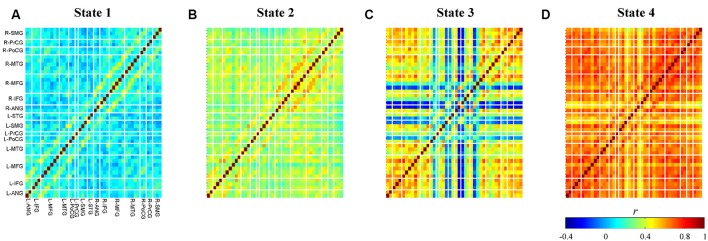
Obtained centroid maps for each cluster **(A–D)**. Colors represent connectivity strength (*r*) between two channels. Channels are categorized and ordered according to spatially registered regions (L, left; R, right; ANG, angular gyrus; IFG, inferior frontal gyrus; MFG, middle frontal gyrus; MTG, middle temporal gyrus; PoCG, post-central gyrus; PrCG, pre-central gyrus; SMG, supramarginal gyrus; STG, superior temporal gyrus).

The relationship between cluster numbers and explained variance is shown in Figure 5. As expected, a non-linear positive relationship was obtained. While two clusters could only address the explained variance of about 60%, unsupervised clustering with 10 clusters accommodated explained variance of approximately 93%. The “elbow” might not be empirically determined; thus, the optimum cluster number was selected on the basis of the longest distance between a first-order line (clusters two and 10) and other points. If there were two or more cluster numbers revealing a relatively similar distance, the less cluster number was preferred to minimize the risk of overfitting. Four clusters with an explained variance of 82% was selected as the optimum cluster number. Four centroid maps are shown in Figure 6. The centroid maps of clusters two and three are the least correlated (see Figures 6B,C; Pearson’s correlation; *r* = 0.40), whereas the centroid maps of clusters one and two are the most correlated (see Figures 6A,B; Pearson’s correlation; *r* = 0.85).

### Connectivity States

**Figure 7:**
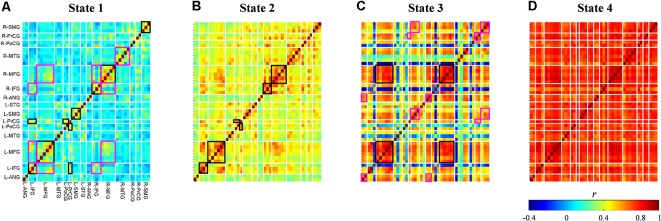
Dynamic functional connectivity maps (Fisher’s *z* transform) averaged across subjects and signal types for each connectivity state **(A–D)**. Colors represent connectivity strength ($\bar{r}$) between two channels. Channels are categorized and ordered according to spatially registered regions (L, left; R, right; ANG, angular gyrus; IFG, inferior frontal gyrus; MFG, middle frontal gyrus; MTG, middle temporal gyrus; PoCG, post-central gyrus; PrCG, pre-central gyrus; SMG, supramarginal gyrus; STG, superior temporal gyrus). Black and magenta rectangles identify regions with strong connectivities in the entire regions and in the several channels of regions, respectively.

Averages of within-cluster connectivity (Fisher’s *z* transformed) for each state are shown in Figure 7. The five features highlighted from these results are explained as follows. First, the dynamic FC of state 1 shows specifically strong within-region connectivities in the bilateral IFG, bilateral MFG, bilateral supramarginal gyrus (SMG), and between-region connectivities in the left precentral gyrus (PrCG) and left postcentral gyrus (PoCG) and left IFG and left PrCG (Figure 7A; black-squared regions). Several channels within the right MFG and left IFG also reveals strong connectivities to channels in the regions of right IFG and left MFG (Figure 7A; magenta-squared regions). Furthermore, the strong within-region connectivity is observed in major channels of the right middle temporal gyrus (MTG) (Figure 7A; magenta-squared regions). Second, the dynamic FC pattern of state 2 (Figure 7B) is similar to that of state 1 (Figure 7A; Pearson’s correlation; *r* = 0.87); however, the range of connectivity strength is stronger in the dynamic FC of state 2 (0.22–0.77 vs. −0.07–0.62). The significantly strong connectivities are found in the within-region of bilateral IFG – MFG and the between-region of left PrCG and left PoCG (Figure 7B; black-squared regions). Third, the dynamic FC of state 3 visualizes the strong (within- and between-region) connectivities in the midline vertex along the bilateral frontal to parietal cortices, such as the bilateral MFG, inter-hemispheric MFG (Figure 7C; black-squared regions), the upper part of bilateral and inter-hemispheric PrCG, SMG, and angular gyrus (ANG) (Figure 7C; magenta-squared regions). Fourth, similar to centroid maps, averages of within-cluster connectivity for states 2 and 3 are the least correlated (c.f. Figures 7B,C; Pearson’s correlation; *r* = 0.34). The low correlation between two connectivity states may suggest differences of connectivity pattern. For example, strong connectivities between bilateral MTG and intra-hemispheric IFG were observed in the connectivity state 2. On the contrary, the connectivity state 3 revealed stronger connectivities of intra-hemispheric ANG and SMG. Fifth, the dynamic FC of state 4 (Figure 7D) represents massive and strong connectivity between all bi-hemispheric channels.

### Characteristics of Connectivity State

**Figure 8:**
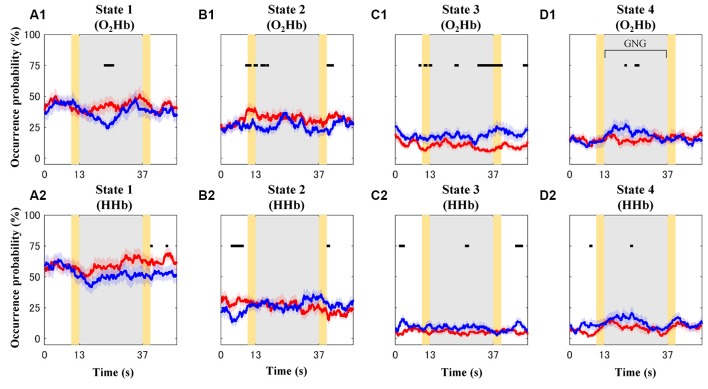
Mean values of occurrence probability for each connectivity state [**A–D** for connectivity states 1–4; (**A1–D1)** and **(A2–D2)** for dynamic O_2_Hb and HHb functional connectivities, respectively] in the case of the typically developing (TD; red plots) and ADHD (blue plots) groups. Gray and yellow patches represent the GNG block as stimulus intervals and the instruction intervals, respectively. Shaded red and blue patches around solid plots indicate within-group standard error. Black squares show significantly different occurrence probability between the TD and ADHD groups at particular time points.

Occurrence probability for each connectivity state (O_2_Hb and HHb; Figures 8A1–D1,A2–D2) and group (TD and ADHD; red and blue plots) is shown in Figure 8. For the visualization purpose only, the plots were represented by mean values of occurrence probability across subjects. However, statistical tests were performed to test median values of occurrence probability between four states, two groups, and two signal types. The four features drawn from these results are explained as follows.

First, the highest occurrence probability was achieved in state 1 (mean of 56%; median of 67%; Dunn-Šidák *post hoc* analysis of Kruskal–Wallis *H* test; $\chi_{\left(3,160\right)}^{2}$ = 97.5; *p* < 10^−20^) for the dynamic HHb FC (Figure 8A2). For the dynamic O_2_Hb FC, the occurrence probabilities of states 1 and 2 (Figures 8A1,B1; means of 40% and 29%; medians of 50% and 33%, respectively) were significantly higher (Dunn-Šidák *post hoc* analysis of Kruskal–Wallis *H* test; $\chi_{\left(3,160\right)}^{2}$ = 51.7; *p* < 10^−10^) than those of states 3 and 4 (Figures 8C1,D1).

Second, there was no significant difference between the occurrence probability of states 3 (mean of 7%–15%; median of 0%–17%) and state 4 (mean of 10%–16%; median of 0%–17%; see Figures 8C,D; Dunn-Šidák *post hoc* analysis of Kruskal-Wallis *H* test) for the dynamic O_2_Hb and HHb FCs.

Third, the occurrence probabilities of states 3 and 4 for the dynamic O_2_Hb FC were significantly higher than those for the dynamic HHb FCs (see Figures 8C1,C2,D1,D2; Wilcoxon signed-rank test; *z* = 2.95–3.08; *p* < 0.01).

Fourth, the occurrence probability of state 1 for the dynamic HHb FC was significantly higher than that for the dynamic O_2_Hb FC (see Figures 8A1,A2; Wilcoxon signed-rank test; *z* = 4.80; *p* < 10^−6^).

These four points suggest that the connectivity states (Friedman test; $\chi_{\left(3\right)}^{2}$ = 145; *p* < 10^−30^) significantly affect occurrence probability. There was no evidence of the effect of signal type *per se* on occurrence probability (Friedman test; $\chi_{\left(1\right)}^{2}$ = 1.65; *p* > 0.05). The statistical results are summarized in Table 1.

**Table 1:** Summary of occurrence probability for each connectivity state and statistical results of occurrence probability comparisons.

Signal type	State 1 (S1)	State 2 (S2)	State 3 (S3)	State 4 (S4)	Inter-state
O_2_Hb	$\bar{x}$ = 40%	$\bar{x}$ = 29%	$\bar{x}$ = 15%	$\bar{x}$ = 16%	S1~S2 > S3~S4
	$\tilde{x}$ = 50%	$\tilde{x}$ = 33%	$\tilde{x}$ = 17%	$\tilde{x}$ = 17%	$\chi_{\left(3,160\right)}^{2}$ = 51.7; *p* < 10^−10^
HHb	$\bar{x}$ = 56%	$\bar{x}$ = 26%	$\bar{x}$ = 7%	$\bar{x}$ = 10%	S1 > S2 > S3~S4
	$\tilde{x}$ = 67%	$\tilde{x}$ = 17%	$\tilde{x}$ = 0%	$\tilde{x}$ = 0%	$\chi_{\left(3,160\right)}^{2}$ = 97.5; *p* < 10^−20^
O_2_Hb vs. HHb	${\text{S1}}_{{\text{(O}}_{\text{2}}\text{Hb)}}{\text{< S1}}_{\text{(HHb)}}$	${\text{S2}}_{{\text{(O}}_{\text{2}}\text{Hb)}}{\text{\textasciitilde{} S2}}_{\text{(HHb)}}$	${\text{S3}}_{{\text{(O}}_{\text{2}}\text{Hb)}}{\text{> S3}}_{\text{(HHb)}}$	${\text{S4}}_{{\text{(O}}_{\text{2}}\text{Hb)}}{\text{> S4}}_{\text{(HHb)}}$
	*z* = −4.80	*z* = 1.65	*z* = 2.95	*z* = 3.08
	*p* < 10^−6^	*p* > 0.1	*p* < 0.01	*p* < 0.01

$\bar{x}$: mean and $\tilde{x}$: median

### Occurrence Probabilities of Connectivity States for TD and ADHD Children

The effects of groups were found at different intervals for each connectivity state. For example, the ADHD group showed significant high occurrence probability (Wilcoxon rank-sum test; *z* = 1.96–3.18; *p* < 0.05) of states 3 (Figure 8C) and 4 (Figure 8D) at some points in the baseline, stimulus, and post-stimulus intervals. However, the ADHD group showed significant low occurrence probability of state 1 after the stimulus onset (blue plot in Figure 8A1; Wilcoxon rank-sum test; *z* = −2.76 to −1.99; *p* < 0.05) and in the post-stimulus interval (blue plots in Figure 8A2; Wilcoxon rank-sum test; *z* = −1.99; *p* < 0.05). The occurrence probabilities of state 2 for the dynamic O_2_Hb and HHb FCs differed in the case of both groups. Significantly increased occurrence probabilities of state 2 (dynamic O_2_Hb FC) were found at the baseline, transition of baseline-to-stimulus, and post-stimulus intervals for the TD group (Figure 8B1; Wilcoxon rank-sum test; *z* = 1.96–2.68; *p* < 0.05). On the other hand, occurrence probability of state 2 for the dynamic HHb FC in the case of the ADHD group (blue plot in Figure 8B2) significantly decreased at the baseline interval (Wilcoxon rank-sum test; *z* = −2.46 to −2.97; *p* < 0.05), but it increased at the end of the stimulus near the post-stimulus interval (Wilcoxon rank-sum test; *z* = 2.10–2.26; *p* < 0.05).

## Discussion

In the current study, fNIRS signals were used for task-based dynamic FC analysis for the first time to the best of our knowledge. Without making any assumptions, we optimized the number of connectivity states. Four different connectivity states were found to shift while the GNG task was performed. The difference between connectivity states was caused by region-specific networks and their occurrence probabilities. A dominant connectivity state was frequently observed (averaged occurrence probability of 40%–56%). Occurrence probabilities of connectivity state dynamically fluctuated, but these fluctuations differed for the TD and ADHD groups. Furthermore, the chances of occurrence probability were seemingly stimulus-dependent. This result may explain atypical ADHD connectivity recruitment and be potentially useful as screening biomarkers.

### Interpretations of Connectivity States

The current results of dynamic FC analysis show four alternating connectivity states during the execution of the GNG task. Even though connectivity states seemingly changed in random ways, strong connectivities within bilateral frontoparietal networks were apparently found in two dominant connectivity states (i.e., states 1 and 2). The frontoparietal network has been associated with attention control (Peers et al., 2005) showing extended activation of the inferior parietal cortex (including SMG and ANG) to accommodate attentional flexibility (Dodds et al., 2011). Since inhibitory control is a complex cognitive process, attention control may also be involved in sequential processes. Successful inhibition response should be able to recognize inhibitory cues that also require intact attention control (Verbruggen et al., 2014; Hong et al., 2017). Moreover, attentional control sometimes correlates with behavioral performances such as omission-commission errors and response time (Murphy et al., 1999; Keilp et al., 2005; Reynolds et al., 2006). According to the above arguments, the relation between the frontoparietal network and complex cognitive processes of inhibition response is justifiably inferred (Dodds et al., 2011; Erika-Florence et al., 2014). It is therefore assumed that both dominant connectivity states are task-related states.

The differences between the two dominant connectivity states are still unclear unless the connectivity of state 2 was stronger and less frequently occurred than those of state 1. The framework of intrinsic-transient networks may explain the two connectivity states. Intrinsic networks should be at least maintained, whereas task-related (i.e., transient) networks may be associated with task demands (Braun et al., 2015). Transient networks are mainly constructed from intrinsic networks (Cole et al., 2014). However, we could not confirm that connectivity state 1 is a task-related intrinsic or transient network based only on connectivity strength and occurrence probability. Furthermore, in the current study, segregation of dynamic FCs for the Go and GNG blocks was insignificant. Even though previous studies evidenced functional differentiation within networks (Menon et al., 2001; Hong et al., 2017), the difficulty of functional differentiation has also been reported (Verbruggen and Logan, 2008). Our recent study also showed that the transitioned connectivity between baseline and stimulus intervals remained subtle particularly in the TD children compared to the ADHD children (Sutoko et al., 2019a).

The frontoparietal network is not specifically involved in the two other connectivity states (states 3 and 4). Thus, those two connectivity states are assumed to be task-irrelevant states. Strong connectivity affecting all regions (i.e., global) was detected in connectivity state 4. A global effect is propagated from cerebral vessels to the local regulation of neurovascular coupling (Roy and Sherrington, 1890; Devonshire et al., 2012). Furthermore, connectivity state 3 shows strong connectivities in overlapped DMN-related regions, particularly in the bilateral medial PFC and ANG (Raichle et al., 2001; Raichle and Snyder, 2007). However, it should be noted that the signal power was concentrated in the low-frequency range: the task-related frequency of interest (0.01–0.8 Hz) was wider than the usual low-frequency oscillations of DMN (<0.2 Hz). While the frontoparietal network is referred to as a task-positive network, the DMN network behaves in an opposite manner, showing deactivation of a task-negative network (Fox et al., 2005). The low occurrence probability of connectivity state 3 (7%–15% on average) happens during task performance, and it might be considered as the compensatory act of increased recruitment of task-positive networks (Cole et al., 2014).

Differences between occurrence probabilities for the O_2_Hb and HHb dynamic FCs in connectivity states 1, 3, and 4 were observed. These differences might be caused by underlying characteristics of systemic effects (Franceschini et al., 2003) and blood-related change sensitivity (Hoshi et al., 2001; Hirasawa et al., 2015). Moreover, frequency-specific connectivity was also non-uniform. High coherence of HHb signals was found in a lower-frequency range than that of the O_2_Hb signals (Sasai et al., 2011). Furthermore, as shown by the datasets used in this study, HHb signals have often been reported to have low SNR (Sasai et al., 2011; Fishburn et al., 2014). The current analysis (in which more HHb signals with low SNR were removed, and the clustering of connectivity state was approximated from remaining signals) may intensify these differences. Therefore, different O_2_Hb and HHb FC behaviors are quite well-grounded.

### Differences Between TD and ADHD Children

The current dynamic FC analysis offers a different perspective in defining differences between TD and ADHD children. Significant group effects were found to interlock with stimulus information, namely, baseline, stimulus, and post-stimulus intervals. Therefore, the task demand may suggest the mechanism of shifting states. The decreased occurrence probability of connectivity state 1 was shown by the ADHD children during the stimulus. Since the frontoparietal network is involved in connectivity state 1, this result may imply decreased effectiveness of task-related connectivity state recruitment in ADHD children. ADHD children revealed lower activation in the right MFG/IFG compared to the activation of the TD children during the GNG task (Monden et al., 2012b; Nagashima et al., 2014b). A meta-analysis of inhibitory control studies (i.e., GNG and SST) found extensive hypoactivation in the supplementary motor area (SMA), ACC, and striato-thalamic areas. This finding suggests impaired right-hemispheric fronto-basal ganglia networks (Hart et al., 2013). A further study is required to reveal relationships between hypoactivation and abnormal state recruitment during the stimulus.

Occurrence probability of connectivity state 2 significantly increases during the transitions between the baseline-stimulus and stimulus-post intervals, particularly in the case of TD children. The transition periods included 3-s instructions on performing the Go and GNG blocks. Therefore, the relationship between task-related state recruitment and task switching may be presumed. The essential role of the medial frontal cortex (including SMA/pre-SMA) in task switching has been demonstrated (Rushworth et al., 2002). Hypoactivation of ADHD children in the SMA/pre-SMA suggested task switching dysfunction (Tamm et al., 2004). Connectivity state 2 comprises strong connectivity between SMA/pre-SMA and other regions aligned within frontoparietal networks. Therefore, the change in occurrence probability of connectivity state 2 likely indicates a temporary condition of task switching that may be lacking in ADHD children.

Occurrence probabilities of assumed task-irrelevant states (i.e., connectivity states 3 and 4) were significantly higher in the case of the ADHD children than those in the TD children during sparse temporal task course. It was reported that the relationship between DMN connectivity and ADHD severity is positively correlated (van Rooij et al., 2015). That report addressed the incapability of ADHD subjects to suppress task-irrelevant networks during inhibition responses. Not only task-specific networks but also other networks (e.g., frontostriatal, fronto-default, and sensory-motor networks) and its interactions among networks had been reported to have dysfunctionalities in ADHD subjects (Castellanos et al., 2006, 2008; Mennes et al., 2012; Choi et al., 2013). Both previous findings and our current results may propound the hypothesis of atypical dynamic state recruitment in ADHD children due to the incapability in accommodating task demands.

### Advantages and Limitations

fNIRS measurement and task-based dynamic FC analysis simultaneously brought two advantages. First, the important finding of atypical dynamic state recruitment could be acquired from practical fNIRS measurement. In the current dataset, no subject was excluded from the analysis. Only 1.11 ± 1.69% of total data points were affected by spikes, and those spikes were corrected by a minimum amount of effort. Therefore, fNIRS provides superior data quality for children measurement compared to fMRI with 30%–50% failure rate (Vaidya et al., 1998; Durston et al., 2003). Second, the task-based dynamic FC analysis offers a controlled measurement environment and sensitive detection of connectivity state. While shifting connectivity states during the RS task may indicate numerous explanations of the conscious level (Barttfeld et al., 2015), performing a task controls common task-related and task-irrelevant connectivity states. Furthermore, a connectivity map was constructed from short signals (12 s) and could be regarded as one of the connectivity states. Therefore, shorter measurement time is feasibly performed, and subject inconveniences can be minimized. Besides confirming our hypothesis of atypical dynamic state recruitment, the current study highlighted clinical benefits. Since the characteristics of hypoactivation and static FC have been explored with the aim of discriminating between ADHD and TD children (Hart et al., 2014; Ishii-Takahashi et al., 2014; Monden et al., 2015; Sutoko et al., 2019a), evaluating the feasibility of dynamic-state recruitment behavior as screening biomarkers will be promising.

In spite of the current findings concerning task-based dynamic FCs and differences between TD-ADHD characteristics, the following two limitations of the current results should be addressed. First, computation methods are still confounding. Effects of SWC-analysis parameters, such as window length, offset, and edge treatment (e.g., tapered weight), have been reported (Shakil et al., 2016). Inappropriate selection of parameters would mislead findings. We tried different window lengths (e.g., 25, 50, and 100 s), and found that using longer window lengths did not significantly improve the explained variance for clustering (82%–83%; data not shown). Similar centroid maps were also obtained across different window lengths (*r* = 0.58–0.89). Even though the clustering analysis is reasonably robust, we have yet to confirm whether the selected window length was appropriate enough for the current task paradigm. The effect of stimulus lengths on window lengths needs to be addressed. Besides the SWC analysis, hidden Markov model has been proposed to reveal the probabilistic model of connectivity state (Vidaurre et al., 2017). There are still opportunities for further improvements in the analysis method. Second, the sample number was small, and the IQ characteristics of the TD and ADHD groups were unmatched. In previous studies, brain hypoactivation is commonly associated with ADHD neuropathophysiology due to good correlation between brain activation, symptomatic ADHD score (Rubia et al., 2005), and behavioral performance (Tamm et al., 2004). Therefore, the current findings should be verified by using a bigger dataset. The effects of fundamental traits (e.g., age, IQ and symptomatic score) and behavioral performances (e.g., omission/commission error and response time) on connectivity states (e.g., occurrence probability and FC strength) should be analyzed further.

## Conclusion

To the best of our knowledge, the current study is the first to use fNIRS signals for investigating task-based dynamic FCs. Connectivity states shifted in alternate ways during the temporal task course. Four connectivity states, which likely represent two dominant task-related states and two task-irrelevant states, were found. Furthermore, the characteristics of TD and ADHD children could be distinguished by the occurrence probability of connectivity states. In the case of ADHD children, the occurrence probability of the dominant connectivity state decreased, and that of the assumed task-irrelevant states within the stimulus interval increased. These results provide a new perspective in explaining ADHD neuropathophysiology related to atypical dynamic state recruitment and promote clinical benefits.

## Data Availability Statement

All datasets generated for this study are included in the article.

## Ethics Statement

The studies involving human participants were reviewed and approved by Ethics committees of Jichi Medical University, the International University of Health and Welfare, Chuo University, and Hitachi, Ltd. Written informed consent to participate in this study was provided by the participants’ legal guardian/next of kin.

## Author Contributions

YM, MN, TI, and TT designed and performed the experiments. SS conceived the presented idea and performed the computation analysis. MK, HA, TT, and ID verified the analysis. YM, AM, TF, TY, and ID supervised the finding of this work. SS wrote the manuscript and ID elaborated it. All authors discussed the results and contributed to the final manuscript.

## Conflict of Interest

SS, TF, HA, MK, and AM are employed by the Hitachi. Ltd. The remaining authors declare that the research was conducted in the absence of any commercial or financial relationships that could be construed as a potential conflict of interest.
